# Supplementary material for: Quantifying climate change impacts emphasises the importance of managing regional threats in the endangered Yellow-eyed penguin
Source: PeerJ. 2017 May 16;5:e3272. doi: 10.7717/peerj.3272 (PMC5436559; doi:10.7717/peerj.3272)
Supplement: Supplemental Information 1 [file peerj-05-3272-s001.docx]

## Electronic Supplementary Material 1.

## Estimation of demographic parameters

Using banding records of Yellow-eyed penguins, we developed a Bayesian mark-recapture (MR) model that was structured into 2 stages: (1) fledglings (age: 0 years) and (2) adults (1+ years). The MR model could not be sex-specific, because information on the gender was reported for only 58% of the individuals in the banding table. The only way to identify individuals were bands which are applied to chicks shortly prior to fledging. This means that our MR model did not consider chicks that died before they could be banded. Overall a total number of 2,967 individuals recorded over a 34-year period were used for analysis.

For the analysis we converted the data into two m-arrays for (1) individuals that were banded as fledglings and recaptured as adults, and (2) individuals that were banded as adults and recaptured as adults (ESM2). Both m-arrays are 2-dimensional matrices providing the number of recaptured individuals in any year (columns) following any release year (rows) ([Burnham 1987](#_ENREF_1)). These m-arrays were analysed using the multinomial distribution, and parameters stage-specific survival *ϕ_k,t_*, with *k* = fledglings or adults in year *t*, and adult recapture ([Kéry and Schaub 2012](#_ENREF_6); [Lebreton et al. 1992](#_ENREF_7)). We fixed the adult recapture probability to one, because the analysed data do not contain reliable recovery records, unless an individual had died or not returned to the breeding colony.

We allowed survival to be time-variant by modelling *ϕ_k,t_* on logit scale, which we assume to vary following a normal distribution with mean zero and stage-specific variance *σ_k_^2^*:

logit(*ϕ_k,t_*) = *μ_k_* + ε_k,t_ [E1.1]

ε_k,t_ ~ Normal(0, *σ_k_^2^*) [E1.2]

where ε_k,t_ denotes the temporal residuals for *μ_k_*. We supplied a uniform prior distribution on the interval [0, 1] for the mean survival on probability scale ($\bar{\phi}$*_k_*), which was linked to *µ_k_* using the link function *µ_k_* = log($\bar{\phi}_{k}$/(1-$\bar{\phi}_{k}$)). We supplied uniform prior distributions for *σ_k_* on the three alternative intervals for [0, 2], [0, 5], and [0, 10], and found no prior sensitivity for estimated *σ_k_ ^2^* (ESM3).

Moreover, we extended the model described in eq. E1.1 to examine the effect of environmental covariates on *ϕ_k,t_*:

logit(*ϕ_k,t_*) = *μ_k_* + β_k_*x_t_ + ε_k,t_ [E1.3]

where *β_k_* denotes the stage-specific effect size fitted to the time-specific covariate x_t_. Except for covariates reflecting SST anomaly we standardized ((x_t_ - $\bar{x}$_t_) / sd(x_t_)) all covariates before model fitting. To ensure that the data provide sufficient information to estimate each parameter, we assessed correlation matrices for each model parameterization (ESM4) ([Gimenez et al. 2008](#_ENREF_4)), which was generally low (range [-0.14, 0.14]), although moderate cross correlation existed between the posterior estimates for mean survival and the effect size (range [-0.38, -0.21]) when we fitted models to covariates reflecting sst anomaly (ESM4). However, we found no sensitivity of estimates for *β_k_* after varying the standard deviation of the normal prior supplied for *β_k_* (N(0, 0.001) and N(0, 0.01)) (ESM4). Additional prior configurations were imposed during the model selection procedure (see below).

For all models (eq. E1.1 and eq. E1.3) we found no indication for a lack-of-fit, based on Bayesian p-values ([Gelman 2013](#_ENREF_2); [Gelman et al. 1996](#_ENREF_3)) that ranged between 0.28 and 0.48.

The model was analysed using JAGS ([Plummer 2003](#_ENREF_9)). The burn-in was 2,000 iterations followed by 25,000 iterations, and posterior samples were drawn using a thinning interval of 3. We computed the potential scale reduction factor $\hat{R}$(Gelman and Rubin 1992) using the output of three MCMC chains and assumed convergence if $\hat{R}$was near to 1. For all estimated parameters $\hat{R}$̂ was smaller than or equal to 1.01.

##### Model selection

First, we fitted the model described in eq. 3 to each covariate separately and computed the percentage of temporal variance explained by each covariate ([Grosbois et al. 2008](#_ENREF_5)) as:

((σ_k_^2^ - σ_k,x_^2^) / σ_k_^2^) * 100 [E1.4]

where σ_k_^2^ and σ_k,x_^2^ are the temporal variance of the stage-specific survival from eq. E1.1 and eq. E1.3, respectively. We considered covariates as influential predictors if they explained at least 20% of σ_k_^2^ ([Grosbois et al. 2008](#_ENREF_5)). For each stage, the two strongest predictors were implemented into the MR model (i.e. eq. E1.3 plus an additional β_k_ for the second covariate in each stage). We assessed the posterior model probability for each combination of these four covariates using Gibbs Variable Selection (GVS) (Ntzoufras 2002; Tenan et al. 2014; Hooten and Hobbs 2015). Importantly, we repeated the analysis with various prior distributions and found that our results were not affected by the choice of priors required for GVS (ESM5).

## Population model

To model YEP population dynamics we developed a female-only model assuming a birth-pulse population ([Tang and Chen 2002](#_ENREF_10)). Due to the lack of consistent data on gender in the database, we followed Richdale (1952) and assumed the sex ratio in YEP to be equal at hatching. As pointed out above, the MR model being based on banding information could not incorporate chick survival/fecundity. For the population model we accounted for this by determining the proportion of female chicks that hatched and remained within the population until fledging. We denoted this as

$\bar{\gamma}$*_chicks_* $=\bar{\rho}* \bar{\omega}$ ** 0.5* [E1.5]

where $\bar{\rho}$ is the average proportion of breeding adults derived from the data of all banded adults (i.e. breeders vs. non-breeders with an age of 1+ years). $\bar{\omega}$ denotes the average proportion of chicks per nest, which was obtained from annual nest monitoring at Boulder Beach*.* To ensure that only female individuals enter the model, we multiplied $\bar{\omega}$ by 0.5 (i.e. based on a 1:1 sex ratio).

After leaving the nest in March, fledglings only have to survive 7 months (on average 212.971 days) until they can be recaptured again at the beginning of the following nesting season in November. Because of this we rescaled the fledgling survival from our MR model to reflect survival over 12-month time interval:

*Φ_fledgling t_* = *ϕ_fledglings,t_*^(1/(365/212.917))^  [E1.6]

Using this information we can now describe γ*_adult,t_*, the proportion of adult YEPs within the population in year *t* as:

$\gamma_{adult,t}= \phi_{adults,t}+ \phi_{fledgling,t}* \bar{\gamma}$*_chicks_*  [E1.7]

where $\phi_{adult,t}\mathrm{and}\phi_{fledgling,t}$ are the only time-varying components, which were derived from posterior distributions for *ϕ_fledglings,t_* and *ϕ_adults,t_* from the model in eq. E1.3 using the covariates that were preserved during the model selection exercise.

For the study years 1982 to 2014 we projected *n_adult,t+1_*, the number of adult YEPs in the year *t+1* as:

n*_adult,t+1_* = n*_adult,t_* * γ*_adult,t_* [E1.8]

where the initial adult population size at t=1 was calculated as:

n*_adult,1_* = n*_nest,1_* / $\bar{\rho}$ [E1.9]

where n*_nest,1_* is the observed number of nests in the first year (i.e. 1982) and $\bar{\rho}$ is the proportion of breeders in the same year.

The average population size is determined by the survival parameters, the average proportion of breeders, and the proportion of fledglings per nest (i.e. a proxy for fecundity); the latter two contained in the variable $\bar{\gamma}$*_chicks_*. The temporal fluctuation of n*_adult,t+1_* only depends on the annual survival rates. Therefore, we visually assessed whether the use of the estimated temporal variance in survival is sufficient to predict the observed change in the adult population size, which was calculated through eq. 9 using the year-specific number of nest between 1982 and 2014.

##### Effect of covariates on the population growth rate

The linear function described in eq. E1.7 reflects the proportional change of adult individuals between years and thus the population growth rates from year *t* to *t+1*. Thus, replacing temporal varying components *ϕ_fledgling,t_* and *ϕ_adults,t_* in eq. E1.7 with average survival rates can be used to approximate the deterministic population growth rate λ (λ < 1: population decline, λ = 1: stable population, λ > 1: population growth) between 1982 and 2014:

λ = $\bar{\phi}$*_adults_ +* $\bar{\phi}$*_fledgling_* * $\bar{\gamma}$*_chicks_* [E1.10]

with $\bar{\phi}$*_adults_* and $\bar{\phi}$*_fledgling_* derived from the model described in eq. E1.3. Moreover, we calculated λ after rescaling each $\bar{\phi}$*_k_* using the average sea surface temperature (SST) anomaly (the predictor variable preserved during the model selection exercise; see ‘Results’) (1) between the years 1982 and 1996; (2) between the years 1996 and 2014. This cut-off was based on visual examination of the SST anomaly, which implies that the SST increased above average after 1996.

## Future projections

To assess the future developments of the penguin population we ran a series of stochastic projections based on our population model. Firstly we simulated future SST anomaly developments until 2060 which included the predicted SST rise for the Otago region of 2.0°C between 1990 and 2090 which translates to a mean 0.02°C increase per year ([Ministry for the Environment 2008](#_ENREF_8)). For each future year up until 2065, we firstly randomly selected 12 monthly averages (i.e. Jan-Dec) from the SST data recorded between 1982 and 2015 data to calculate an annual mean. We then cumulatively added 0.02°C to each simulated annual mean (i.e. 2015-2060) and calculated the corresponding SST anomaly as difference of the average annual SST anomaly. We ran 1,000 iterations of this simulation and used the mean of all simulations as the projected SST anomaly, which served to predict future survival rates using eq. 1 and 2 and parameters estimated from the MR data. Each forward prediction of survival rates was repeated 500 times for all posterior samples to allow for uncertainty owing to temporal stochasticity. These predicted survival rates were then used to forward project the adult population size from 2015 onwards. After 2012 it was difficult to determine whether an individual’s absence was due to absence from breeding or actual mortality. To ensure that the future projections are not affected through underestimated survival rates between 2012 and 2014, we re-fitted the MR model to data sets limited to the years 1982-2011, 1982-2012 and 1982-2013; and started each projection in 2012, 2013 and 2014, respectively.

## References

Burnham, K.P., 1987. Design and analysis methods for fish survival experiments based on release-recapture. American Fisheries Society.

Gelman, A., 2013. Two simple examples for understanding posterior p-values whose distributions are far from unform. Electronic Journal of Statistics 7, 2595-2602.

Gelman, A., Meng, X.-L., Stern, H., 1996. Posterior predictive assessment of model fitness via realized discrepancies. Statistica sinica, 733-760.

Gimenez, O., Morgan, B., Brooks, S., 2008. Weak identifiability in models for mark-recapture-recovery data, In Modeling demographic processes in marked populations. eds D.L. Thomson, E.G. Cooch, M.J. Conroy, pp. 1055-1067. Springer Science & Business Media.

Grosbois, V., Gimenez, O., Gaillard, J.M., Pradel, R., Barbraud, C., Clobert, J., Møller, A., Weimerskirch, H., 2008. Assessing the impact of climate variation on survival in vertebrate populations. Biological Reviews 83, 357-399.

Kéry, M., Schaub, M., 2012. Bayesian population analysis using WinBUGS: a hierarchical perspective. Academic Press.

Lebreton, J., Bunham, K., Clobert J, Anderson, D., 1992. Modeling survival and testing biological hypotheses using marked animals - A unified approach with case-studies. Ecological Monographs 62, 67-118.

Ministry for the Environment, 2008. Climate Change Effects and Impacts Assessment: A Guidance Manual for Local Government in New Zealand. 2nd Edition., eds B. Mullan, D. Wratt, S. Dean, M. Hollis, S. Allan, T. Williams, G. Kenny, p. xviii + 149 p. Ministry for the Environment,, Wellington.

Plummer, M., 2003. JAGS: A program for analysis of Bayesian graphical models using Gibbs sampling, In Proceedings of the 3rd international workshop on distributed statistical computing. p. 125. Technische Universit at Wien Wien, Austria.

Tang, S., Chen, L., 2002. Density-dependent birth rate, birth pulses and their population dynamic consequences. Journal of Mathematical Biology 44, 185-199.
