# Supplementary material for: Quantifying climate change impacts emphasises the importance of managing regional threats in the endangered Yellow-eyed penguin
Source: PeerJ. 2017 May 16;5:e3272. doi: 10.7717/peerj.3272 (PMC5436559; doi:10.7717/peerj.3272)
Supplement: Supplemental Information 3 [file peerj-05-3272-s003.pdf]

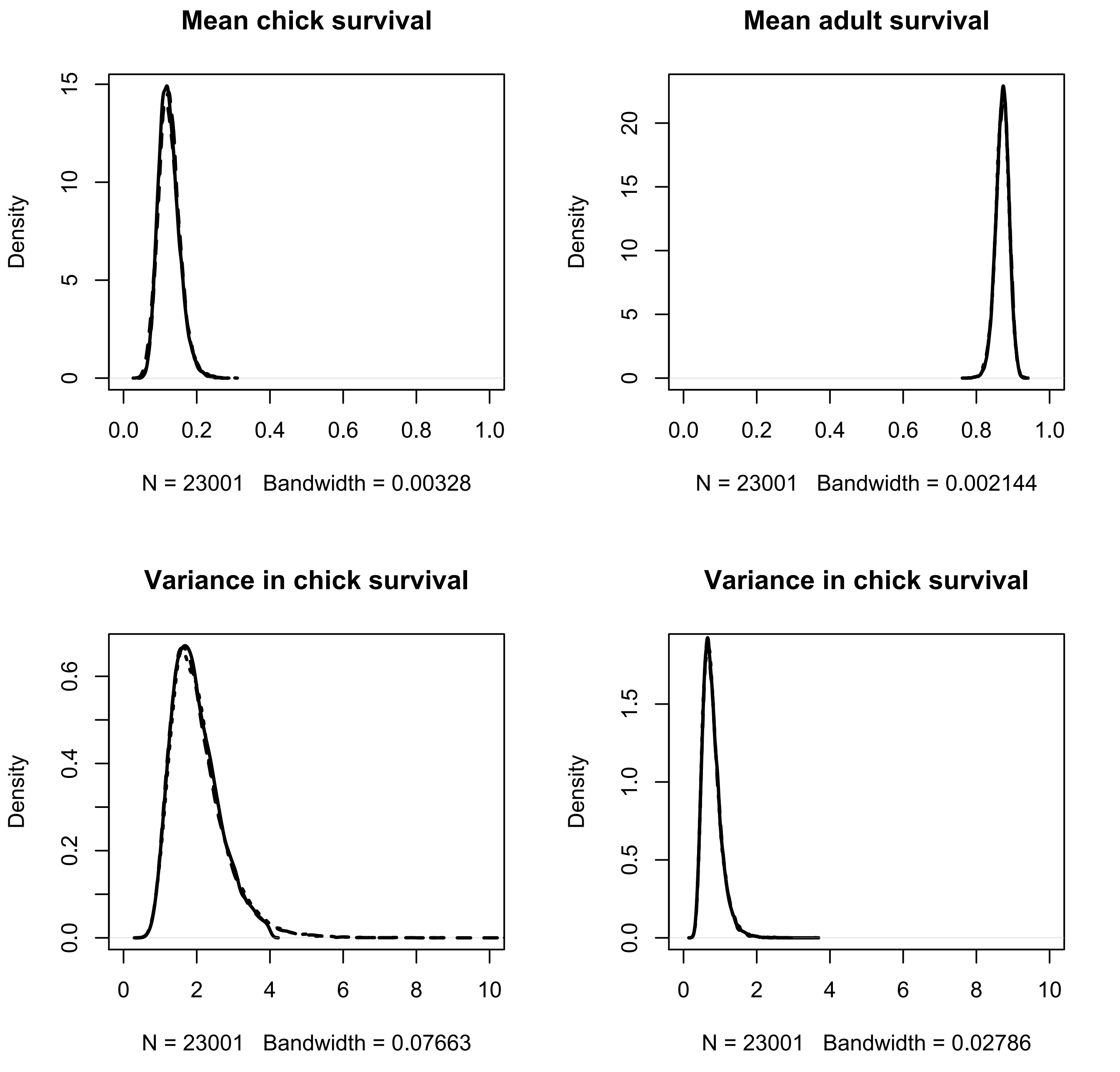

Mean chick survival
Density
N = 23001 Bandwidth = 0.00328
Mean adult survival
Density
N = 23001 Bandwidth = 0.002144
Variance in chick survival
Density
N = 23001 Bandwidth = 0.07663
Variance in chick survival
Density
N = 23001 Bandwidth = 0.02786
