# Supplementary material for: Quantifying climate change impacts emphasises the importance of managing regional threats in the endangered Yellow-eyed penguin
Source: PeerJ. 2017 May 16;5:e3272. doi: 10.7717/peerj.3272 (PMC5436559; doi:10.7717/peerj.3272)
Supplement: Supplemental Information 4 [file peerj-05-3272-s004.pdf]

crosscorr.cjs.yep.daily_min_temp_annual

	beta.a	beta.j	mean.phiad	mean.phijuv	sigma2.a	sigma2.j
beta.a	1	-0.02	-0.12	0.02	-0.05	-0.02
beta.j		1	-0.02	0.07	0	-0.13
mean.phiad			1	-0.05	0.08	0
mean.phijuv				1	0	-0.08
sigma2.a					1	-0.01
sigma2.j						1

crosscorr.cjs.yep.daily_min_temp_mar_may

	beta.a	beta.j	mean.phiad	mean.phijuv	sigma2.a	sigma2.j
beta.a	1	0	-0.07	-0.01	-0.06	-0.02
beta.j		1	0	0.08	0	-0.02
mean.phiad			1	0	0.08	0.01
mean.phijuv				1	0	-0.03
sigma2.a					1	0
sigma2.j						1

crosscorr.cjs.yep.days_wind_gusts_33_annual

	beta.a	beta.j	mean.phiad	mean.phijuv	sigma2.a	sigma2.j
beta.a	1	0	0.08	0.02	0.07	0.01
beta.j		1	0	-0.1	-0.01	0.12
mean.phiad			1	0	0.05	0
mean.phijuv				1	-0.01	-0.08
sigma2.a					1	0
sigma2.j						1

crosscorr.cjs.yep.days_wind_gusts_33_mar_may

	beta.a	beta.j	mean.phiad	mean.phijuv	sigma2.a	sigma2.j
beta.a	1	-0.02	0.06	0.01	0.09	0
beta.j		1	-0.01	-0.07	0	0.14
mean.phiad			1	0.04	0.05	-0.02
mean.phijuv				1	0	-0.05
sigma2.a					1	0
sigma2.j						1

crosscorr.cjs.yep.max_1day_rain_annual

	beta.a	beta.j	mean.phiad	mean.phijuv	sigma2.a	sigma2.j
beta.a	1	-0.01	0.02	0	0.03	0
beta.j		1	0.01	-0.01	0	-0.03
mean.phiad			1	-0.01	0.02	0.01
mean.phijuv				1	0.01	-0.01
sigma2.a					1	0
sigma2.j						1

crosscorr.cjs.yep.max_1day_rain_mar_may

	beta.a	beta.j	mean.phiad	mean.phijuv	sigma2.a	sigma2.j
beta.a	1	0.01	0.04	-0.02	0.03	0.01
beta.j		1	0.02	0.1	0.01	-0.04
mean.phiad			1	0.01	0.03	0.01
mean.phijuv				1	-0.01	-0.07
sigma2.a					1	0
sigma2.j						1

crosscorr.cjs.yep.mean_air_temp_annual

	beta.a	beta.j	mean.phiad	mean.phijuv	sigma2.a	sigma2.j
beta.a	1	0	-0.08	-0.02	-0.09	0
beta.j		1	0.02	0.08	-0.01	-0.14
mean.phiad			1	-0.03	0.06	0
mean.phijuv				1	-0.01	-0.06
sigma2.a					1	0.01
sigma2.j						1

crosscorr.cjs.yep.mean_air_temp_mar_may

	beta.a	beta.j	mean.phiad	mean.phijuv	sigma2.a	sigma2.j
beta.a	1	0.05	-0.07	0	-0.04	0
beta.j		1	-0.02	0.09	-0.01	-0.06
mean.phiad			1	0.01	0.06	0
mean.phijuv				1	0	-0.07
sigma2.a					1	0.01
sigma2.j						1

crosscorr.cjs.yep.sst_anomaly_austral

	beta.a	beta.j	mean.phiad	mean.phijuv	sigma2.a	sigma2.j
beta.a	1	-0.01	-0.38	0.01	-0.06	0.01
beta.j		1	0.01	-0.25	0	-0.2
mean.phiad			1	-0.03	0.06	-0.02
mean.phijuv				1	-0.01	0.03
sigma2.a					1	0
sigma2.j						1

crosscorr.cjs.yep.sst_anomaly_mar_june

	beta.a	beta.j	mean.phiad	mean.phijuv	sigma2.a	sigma2.j
beta.a	1	-0.03	-0.36	0.02	-0.07	-0.01
beta.j		1	0.01	-0.21	0	-0.04
mean.phiad			1	-0.02	0.09	0.01
mean.phijuv				1	-0.01	-0.03
sigma2.a					1	-0.01
sigma2.j						1

crosscorr.cjs.yep.sst_anomaly_minus_1yr

	beta.a	beta.j	mean.phiad	mean.phijuv	sigma2.a	sigma2.j
beta.a	1	0	-0.35	-0.01	-0.06	-0.01
beta.j		1	-0.02	-0.21	-0.02	0
mean.phiad			1	0	0.08	-0.01
mean.phijuv				1	0.01	-0.04
sigma2.a					1	0
sigma2.j						1

crosscorr.cjs.yep.sst_anomaly_minus_2yrs

	beta.a	beta.j	mean.phiad	mean.phijuv	sigma2.a	sigma2.j
beta.a	1	0.01	-0.21	-0.01	0.01	0.01
beta.j		1	0	-0.22	-0.02	0.01
mean.phiad			1	-0.04	0.05	0
mean.phijuv				1	0	0.02
sigma2.a					1	0
sigma2.j						1

crosscorr.cjs.yep.total_rainfall_annual

	beta.a	beta.j	mean.phiad	mean.phijuv	sigma2.a	sigma2.j
beta.a	1	-0.01	0	0.01	0.03	0.01
beta.j		1	0.01	0.02	0	-0.06
mean.phiad			1	-0.03	0.04	-0.01
mean.phijuv				1	0	-0.02
sigma2.a					1	0
sigma2.j						1

crosscorr.cjs.yep.total_rainfall_mar_may

	beta.a	beta.j	mean.phiad	mean.phijuv	sigma2.a	sigma2.j
beta.a	1	0	0.01	0.02	0.06	-0.01
beta.j		1	0.01	0.09	0.01	-0.05
mean.phiad			1	0	0.02	0
mean.phijuv				1	-0.01	-0.05
sigma2.a					1	0
sigma2.j						1

crosscorr.cjs.yep.wet_days_annual

	beta.a	beta.j	mean.phiad	mean.phijuv	sigma2.a	sigma2.j
beta.a	1	0	0.01	-0.02	0.01	-0.02
beta.j		1	0.03	-0.04	0.02	0.06
mean.phiad			1	0.01	0.06	0
mean.phijuv				1	0.02	-0.02
sigma2.a					1	-0.01
sigma2.j						1

crosscorr.cjs.yep.wet_days_mar_may

	beta.a	beta.j	mean.phiad	mean.phijuv	sigma2.a	sigma2.j
beta.a	1	-0.01	-0.01	0.02	0	-0.01
beta.j		1	0.01	0.04	0	-0.01
mean.phiad			1	0	0.07	0
mean.phijuv				1	0	0
sigma2.a					1	0.01
sigma2.j						1

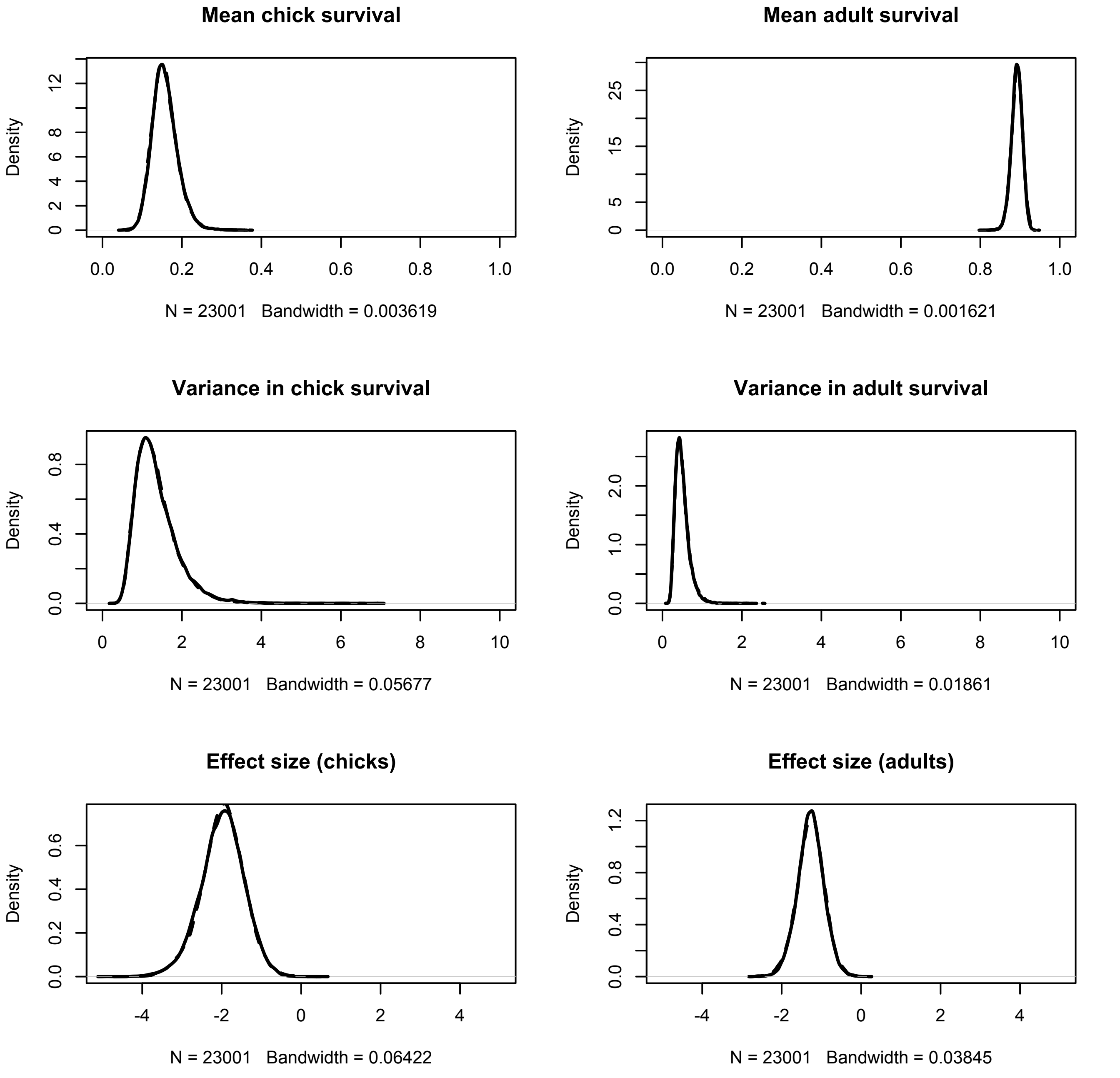
Mean chick survival
Density
N = 23001 Bandwidth = 0.003619
Mean adult survival
N = 23001 Bandwidth = 0.001621
Variance in chick survival
N = 23001 Bandwidth = 0.05677
Variance in adult survival
N = 23001 Bandwidth = 0.01861
Effect size (chicks)
N = 23001 Bandwidth = 0.06422
Effect size (adults)
N = 23001 Bandwidth = 0.03845
