# Supplementary material for: Quantifying climate change impacts emphasises the importance of managing regional threats in the endangered Yellow-eyed penguin
Source: PeerJ. 2017 May 16;5:e3272. doi: 10.7717/peerj.3272 (PMC5436559; doi:10.7717/peerj.3272)
Supplement: Supplemental Information 5 [file peerj-05-3272-s005.docx]

**Electronic Supplementary Material 5.**

**Estimation of the posterior model probability using Gibbs Variable Selection (GVS)**

The MR model was simultaneously fitted to the covariates ‘sst_anomaly_austral’ and ‘days_wind_gusts_33_annual’ for chick survival (Table 2 in main text) and covariates ‘sst_anomaly_austral’ and ‘mean_air_temp_annual’ for adult survival . We assessed the posterior model probability (p(M_i_|y)) for each combination of these four covariates using Gibbs variable selection (GVS) (Ntzoufras 2002; Tenan et al. 2014; Hooten and Hobbs 2015). For this purpose we modeled survival in stage *k* at time *t* as:

$logit\left( \eta_{k,t} \right)= \mu_{k}+ \sum_{j=0}^{w} \gamma_{j}X_{j,t}\beta_{k,j}$ + ε_k,t_ [E5.1]

where X_j,t_ and β_k,j_ denote the design matrix of covariate *j* in year *t* and the slope parameter *j* for stage *k*, respectively (*w* is the maximum number of covariates considered). $\mu_{k}$ and ε_k,t_ reflect the stage-specific mean survival and random effects (see ESM1 eq. E1.1 and eq. E1.2). γ_j_ denotes the auxiliary indicator variable and is a binary response variable that indicates whether covariate *β_k,j_* is present (γ_j_ = 1) or absent (γ_j_ = 0) in the model. We assumed that the chance that γ_j_ = 1 follows a Bernoulli trial with probability 0.5. To ensure good mixing for indicator γ_j_ and effect variable *β_k,j_* we assumed that both variables depend on each other (Ntzoufras 2002; Tenan et al. 2014) by modeling the prior for *β_k,j_* |(γ_j_ = 1) and a pseudoprior for *β_k,j_* |(γ_j_ = 0) using a mixture prior:

$p\left( \beta_{k,j} | \gamma_{j} \right)=\left( 1- \gamma_{j} \right)Normal\left( \kappa_{j}, S_{j} \right)+\gamma_{j}Normal(0, \Sigma_{j})$ [E5.2]

where $\kappa$*_j_* and *S_j_* are user-defined tuning parameters and Σ_j_ denotes the fixed prior variance for *β_k,j_*. The posterior model probability p(M_i_|y) for each model M_i_ (i = 16 possible models for each combination of the considered covariates) is given by:

p(M_i_|y) = Number of occurrences of M = *i* / Total number of iterations

We followed Tenan *et al.* (2014) and checked whether p(M_i_|y) is sensitive to the mixture prior by repeating the analysis with different priors for $\mathrm{Normal}\left( \kappa_{j}, S_{j} \right)$:(1) $\mathrm{Normal}\left( \bar{\beta_{k,j}}, SD(\beta_{k,j}) \right)$, which is a normal prior for *β_k,j_* with mean and standard deviation taken from the posterior distribution of each *β_k,j_* of separate model runs; (2) N(0, 10); (3) N(0, 100); (4) N(0, 1000); (5) N(0, 10^6^); (6) N(0.2, 100) (Tenan *et al.* 2014).

The burn-in was 1,400,000 iterations followed by 1,500,000 iterations and posterior samples were drawn using a thinning interval 3. We computed the potential scale reducing factor (Gelman and Rubin 1992) using the output of three MCMC chains and assumed convergence if was near to 1. For all estimated parameters was smaller than or equal to 1.01. Overall there exist no sensitivity of the estimated posterior model probability to the set of prior distributions used (Table E5.1-Table E5.6). Prior set (5) did not fully converge, but produced similar results to all other prior configurations (Table E5.5).

**Table E5.1** Posterior model probability p(M_i_|y) using actual posterior distributions estimated for each β_k_. Column A: The MR model considers the covariate sst_anomaly_austral for chick survival; Column B: The MR model considers the covariate days_wind_gusts_33_annual for chick survival; Column C: The MR model considers the covariate sst_anomaly_austral for adult survival; Column D: The MR model considers the covariate mean_air_temp_annual for adult survival.

|  | **Model configuration** | | | |  |
| --- | --- | --- | --- | --- | --- |
| **M_i_** | **A** | **B** | **C** | **D** | **p(M_i_\|y)** |
| 1 | 1 | 0 | 1 | 0 | 0.42 |
| 2 | 0 | 1 | 1 | 0 | 0.13 |
| 3 | 1 | 1 | 1 | 0 | 0.12 |
| 4 | 1 | 0 | 0 | 1 | 0.09 |
| 5 | 0 | 1 | 0 | 1 | 0.06 |
| 6 | 0 | 0 | 0 | 0 | 0.04 |
| 7 | 1 | 0 | 0 | 0 | 0.03 |
| 8 | 0 | 1 | 0 | 0 | 0.03 |
| 9 | 1 | 1 | 0 | 0 | 0.02 |
| 10 | 0 | 0 | 1 | 0 | 0.01 |
| 11 | 0 | 0 | 0 | 1 | 0.01 |
| 12 | 1 | 1 | 0 | 1 | 0.01 |
| 13 | 0 | 0 | 1 | 1 | 0.00 |
| 14 | 1 | 0 | 1 | 1 | 0.00 |
| 15 | 0 | 1 | 1 | 1 | 0.00 |
| 16 | 1 | 1 | 1 | 1 | 0.00 |

**Table E5.2** Posterior model probability p(M_i_|y) using a normally distributed prior for β_k_: N(0, 10). See Column A: The MR model considers the covariate sst_anomaly_austral for chick survival; Column B: The MR model considers the covariate days_wind_gusts_33_annual for chick survival; Column C: The MR model considers the covariate sst_anomaly_austral for adult survival; Column D: The MR model considers the covariate mean_air_temp_annual for adult survival.

|  | **Model configuration** | | | |  |
| --- | --- | --- | --- | --- | --- |
| **M_i_** | **A** | **B** | **C** | **D** | **p(M_i_\|y)** |
| 1 | 1 | 0 | 1 | 0 | 0.35 |
| 2 | 1 | 0 | 0 | 1 | 0.17 |
| 3 | 0 | 1 | 1 | 0 | 0.15 |
| 4 | 1 | 1 | 1 | 0 | 0.07 |
| 5 | 0 | 1 | 0 | 1 | 0.05 |
| 6 | 0 | 0 | 0 | 0 | 0.04 |
| 7 | 1 | 0 | 0 | 0 | 0.04 |
| 8 | 0 | 1 | 0 | 0 | 0.03 |
| 9 | 1 | 1 | 0 | 0 | 0.03 |
| 10 | 0 | 0 | 1 | 0 | 0.02 |
| 11 | 0 | 0 | 0 | 1 | 0.01 |
| 12 | 1 | 1 | 0 | 1 | 0.01 |
| 13 | 0 | 0 | 1 | 1 | 0.00 |
| 14 | 1 | 0 | 1 | 1 | 0.00 |
| 15 | 0 | 1 | 1 | 1 | 0.00 |
| 16 | 1 | 1 | 1 | 1 | 0.00 |

**Table E5.3** Posterior model probability p(M_i_|y) using a normally distributed prior for β_k_: N(0, 100). Column A: The MR model considers the covariate sst_anomaly_austral for chick survival; Column B: The MR model considers the covariate days_wind_gusts_33_annual for chick survival; Column C: The MR model considers the covariate sst_anomaly_austral for adult survival; Column D: The MR model considers the covariate mean_air_temp_annual for adult survival.

|  | **Model configuration** | | | |  |
| --- | --- | --- | --- | --- | --- |
| **M_i_** | **A** | **B** | **C** | **D** | **p(M_i_\|y)** |
| 1 | 1 | 0 | 1 | 0 | 0.43 |
| 2 | 1 | 0 | 0 | 1 | 0.19 |
| 3 | 0 | 1 | 1 | 0 | 0.11 |
| 4 | 1 | 1 | 1 | 0 | 0.06 |
| 5 | 0 | 1 | 0 | 1 | 0.05 |
| 6 | 0 | 0 | 0 | 0 | 0.05 |
| 7 | 1 | 0 | 0 | 0 | 0.04 |
| 8 | 0 | 1 | 0 | 0 | 0.02 |
| 9 | 1 | 1 | 0 | 0 | 0.02 |
| 10 | 0 | 0 | 1 | 0 | 0.01 |
| 11 | 0 | 0 | 0 | 1 | 0.01 |
| 12 | 1 | 1 | 0 | 1 | 0.00 |
| 13 | 0 | 0 | 1 | 1 | 0.00 |
| 14 | 1 | 0 | 1 | 1 | 0.00 |
| 15 | 0 | 1 | 1 | 1 | 0.00 |
| 16 | 1 | 1 | 1 | 1 | 0.00 |

**Table E5.4** Posterior model probability p(M_i_|y) using a normally distributed prior for β_k_: N(0, 1000). Column A: The MR model considers the covariate sst_anomaly_austral for chick survival; Column B: The MR model considers the covariate days_wind_gusts_33_annual for chick survival; Column C: The MR model considers the covariate sst_anomaly_austral for adult survival; Column D: The MR model considers the covariate mean_air_temp_annual for adult survival.

|  | **Model configuration** | | | |  |
| --- | --- | --- | --- | --- | --- |
| **M_i_** | **A** | **B** | **C** | **D** | **p(M_i_\|y)** |
| 1 | 1 | 0 | 1 | 0 | 0.42 |
| 2 | 1 | 0 | 0 | 0 | 0.14 |
| 3 | 0 | 1 | 1 | 0 | 0.13 |
| 4 | 1 | 1 | 1 | 0 | 0.09 |
| 5 | 1 | 0 | 0 | 1 | 0.05 |
| 6 | 0 | 0 | 0 | 0 | 0.04 |
| 7 | 0 | 1 | 0 | 0 | 0.04 |
| 8 | 1 | 1 | 0 | 0 | 0.03 |
| 9 | 0 | 0 | 1 | 0 | 0.02 |
| 10 | 0 | 0 | 0 | 1 | 0.01 |
| 11 | 0 | 1 | 0 | 1 | 0.01 |
| 12 | 1 | 1 | 0 | 1 | 0.01 |
| 13 | 0 | 0 | 1 | 1 | 0.01 |
| 14 | 1 | 0 | 1 | 1 | 0.00 |
| 15 | 0 | 1 | 1 | 1 | 0.00 |
| 16 | 1 | 1 | 1 | 1 | 0.00 |

**Table E5.5** Posterior model probability p(M_i_|y) using a normally distributed prior for β_k_: N(0, 10^6^). See Column A: The MR model considers the covariate sst_anomaly_austral for chick survival; Column B: The MR model considers the covariate days_wind_gusts_33_annual for chick survival; Column C: The MR model considers the covariate sst_anomaly_austral for adult survival; Column D: The MR model considers the covariate mean_air_temp_annual for adult survival.

|  | **Model configuration** | | | |  |
| --- | --- | --- | --- | --- | --- |
| **M_i_** | **A** | **B** | **C** | **D** | **p(M_i_\|y)** |
| 1 | 1 | 0 | 1 | 0 | 0.43 |
| 2 | 1 | 0 | 0 | 1 | 0.13 |
| 3 | 0 | 1 | 1 | 0 | 0.12 |
| 4 | 1 | 1 | 1 | 0 | 0.08 |
| 5 | 0 | 0 | 0 | 0 | 0.06 |
| 6 | 1 | 0 | 0 | 0 | 0.05 |
| 7 | 0 | 1 | 0 | 0 | 0.04 |
| 8 | 1 | 1 | 0 | 0 | 0.03 |
| 9 | 0 | 0 | 1 | 0 | 0.02 |
| 10 | 0 | 0 | 0 | 1 | 0.01 |
| 11 | 0 | 1 | 0 | 1 | 0.01 |
| 12 | 1 | 1 | 0 | 1 | 0.01 |
| 13 | 0 | 0 | 1 | 1 | 0.01 |
| 14 | 1 | 0 | 1 | 1 | 0.00 |
| 15 | 0 | 1 | 1 | 1 | 0.00 |
| 16 | 1 | 1 | 1 | 1 | 0.00 |

**Table E5.6** Posterior model probability p(M_i_|y) using a normally distributed prior for β_k_: N(0.2, 100). Column A: The MR model considers the covariate sst_anomaly_austral for chick survival; Column B: The MR model considers the covariate days_wind_gusts_33_annual for chick survival; Column C: The MR model considers the covariate sst_anomaly_austral for adult survival; Column D: The MR model considers the covariate mean_air_temp_annual for adult survival.

|  | **Model configuration** | | | |  |
| --- | --- | --- | --- | --- | --- |
| **M_j_** | **A** | **B** | **C** | **D** | **p(M_i_\|y)** |
| 1 | 1 | 0 | 1 | 0 | 0.47 |
| 2 | 1 | 0 | 0 | 0 | 0.16 |
| 3 | 0 | 1 | 1 | 0 | 0.11 |
| 4 | 1 | 1 | 1 | 0 | 0.10 |
| 5 | 1 | 0 | 0 | 1 | 0.04 |
| 6 | 0 | 0 | 0 | 0 | 0.03 |
| 7 | 0 | 1 | 0 | 0 | 0.03 |
| 8 | 1 | 1 | 0 | 0 | 0.02 |
| 9 | 0 | 0 | 1 | 0 | 0.02 |
| 10 | 0 | 0 | 0 | 1 | 0.01 |
| 11 | 0 | 1 | 0 | 1 | 0.01 |
| 12 | 1 | 1 | 0 | 1 | 0.00 |
| 13 | 0 | 0 | 1 | 1 | 0.00 |
| 14 | 1 | 0 | 1 | 1 | 0.00 |
| 15 | 0 | 1 | 1 | 1 | 0.00 |
| 16 | 1 | 1 | 1 | 1 | 0.00 |

**References**

Gelman, A., & Rubin, D. B. (1992). Inference from iterative simulation using multiple sequences. Statistical science, 7(4), 457-472.

Hooten, M. B., & Hobbs, N. T. (2015). A guide to Bayesian model selection for ecologists. Ecological Monographs, 85(1), 3-28.

Ntzoufras, I. (2002). Gibbs variable selection using BUGS. Journal of Statistical Software, 7(7), 1-19.

Tenan, S., O’Hara, R. B., Hendriks, I., & Tavecchia, G. (2014). Bayesian model selection: the steepest mountain to climb. Ecological Modelling, 283, 62-69.
